# A Computational Modeling Approach Predicts Interaction of the Antifungal Protein AFP from *Aspergillus giganteus* with Fungal Membranes via Its γ-Core Motif

**DOI:** 10.1128/mSphere.00377-18

**Published:** 2018-10-03

**Authors:** Tillmann Utesch, Alejandra de Miguel Catalina, Caspar Schattenberg, Norman Paege, Peter Schmieder, Eberhard Krause, Yinglong Miao, J. Andrew McCammon, Vera Meyer, Sascha Jung, Maria Andrea Mroginski

**Affiliations:** a Institut für Chemie, Technische Universität Berlin, Berlin, Germany; b Institute of Biotechnology, Department Applied and Molecular Microbiology, Technische Universität Berlin, Berlin, Germany; c Leibniz-Forschungsinstitut für Molekulare Pharmakologie (FMP), Berlin, Germany; d Center for Computational Biology, University of Kansas, Lawrence, Kansas, USA; e Department of Molecular Biosciences, University of Kansas, Lawrence, Kansas, USA; f Department of Chemistry & Biochemistry, University of California, San Diego, La Jolla, California, USA; g Department of Pharmacology, University of California, San Diego, La Jolla, California, USA; Rutgers University

**Keywords:** AFP, antifungal peptides, fungi, membranes, modeling, molecular dynamics, nuclear magnetic resonance

## Abstract

Fungal pathogens kill more people per year globally than malaria or tuberculosis and threaten international food security through crop destruction. New sophisticated strategies to inhibit fungal growth are thus urgently needed. Among the potential candidate molecules that strongly inhibit fungal spore germination are small cationic, cysteine-stabilized proteins of the AFP family secreted by a group of filamentous Ascomycetes. Its founding member, AFP from Aspergillus giganteus, is of particular interest since it selectively inhibits the growth of filamentous fungi without affecting the viability of mammalian, plant, or bacterial cells. AFPs are also characterized by their high efficacy and stability. Thus, AFP can serve as a lead compound for the development of novel antifungals. Notably, all members of the AFP family comprise a γ-core motif which is conserved in all antimicrobial proteins from pro- and eukaryotes and known to interfere with the integrity of cytoplasmic plasma membranes. In this study, we used classical molecular dynamics simulations combined with wet laboratory experiments and nuclear magnetic resonance (NMR) spectroscopy to characterize the structure and dynamical behavior of AFP isomers in solution and their interaction with fungal model membranes. We demonstrate that the γ-core motif of structurally conserved AFP is the key for its membrane interaction, thus verifying for the first time that the conserved γ-core motif of antimicrobial proteins is directly involved in protein-membrane interactions. Furthermore, molecular dynamic simulations suggested that AFP does not destroy the fungal membrane by pore formation but covers its surface in a well-defined manner, using a multistep mechanism to destroy the membranes integrity.

**IMPORTANCE** Fungal pathogens pose a serious danger to human welfare since they kill more people per year than malaria or tuberculosis and are responsible for crop losses worldwide. The treatment of fungal infections is becoming more complicated as fungi develop resistances against commonly used fungicides. Therefore, discovery and development of novel antifungal agents are of utmost importance.

## Abstract (precis)

Fungal pathogens pose a serious danger to human welfare since they kill more people per year than malaria or tuberculosis and are responsible for crop losses worldwide. The treatment of fungal infections is becoming more complicated as fungi develop resistances against commonly used fungicides. Therefore, discovery and development of novel antifungal agents are of utmost importance.

## INTRODUCTION

The small antifungal protein (AFP) from Aspergillus giganteus possesses high potential for fighting pathogenic fungi. It selectively kills human- and plant-pathogenic fungi without affecting mammals or plants. However, before deploying AFP as therapeutic agent, it is crucial to understand its mechanism of action. In our work, we used computer simulations and experiments to investigate the structure and dynamics of AFPs. By simulating AFP's interaction with fungal membranes, we suggest that AFP does not destroy the fungal membrane by pore formation but covers it, forming a “carpet,” and destroys fungal pathogens via a multistep procedure.

Filamentous fungi are of great importance for human welfare. On the one hand, fungal pathogens threaten international food security through crop destruction, are a significant cause of global human morbidity and mortality, and cause ecosystem-level changes to the biosphere. On the other hand, filamentous fungi are a source of high-value products in the biotechnology industry, including antibiotics and therapeutics (1–3). A fundamental process underlying infection is germination of dormant spores. For pathogens, this constitutes a critical phase of disease initiation and host-pathogen interactions that is of crucial importance for infection outcome. Another emerging problem is the evolution of resistant strains due to an overuse of fungicides during recent decades (1, 4, 5). Therefore, the search for new antifungals which selectively inhibit spore germination of fungal pathogens without affecting human and environment is of utmost importance. Remarkably, fungi also represent a rich but thus far untapped resource for developing novel antifungals. Here, antifungal proteins of the AFP family are of special interest. They are small (∼6 kDa), highly stable due to intramolecular disulfide bond formation, exhibit predominant β-sheet structure, adopt an amphipathic three-dimensional (3D) structure with a net cationic charge, and inhibit spore germination via disruption of fungal membrane integrity (of other fungi) without affecting plant or mammalian cell systems (6, 7).

**FIG 1:**
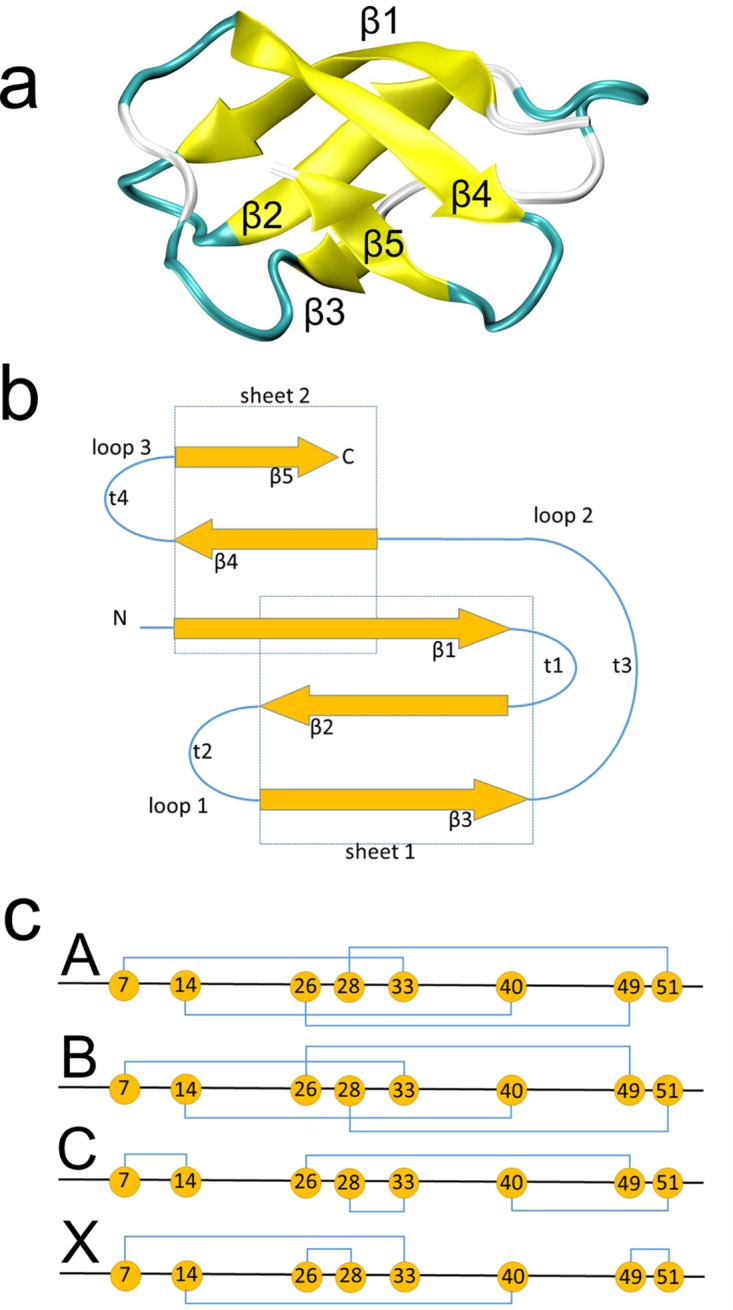
Structure of AFP. The β-barrel structure of AFP (A) and schematic views of the secondary structure (B) and proposed disulfide bridge pattern (C) are shown.

AFP of Aspergillus giganteus is the founder molecule of the AFP family, which consists of more than 50 members (8). The mature protein, which can be found in large amounts in the culture supernatant of A. giganteus, consists of 51 canonical amino acids forming a β–barrel as resolved by nuclear magnetic resonance (NMR) analysis in 1995 (Fig 1A) (9). The five β strands are arranged in two sheets as illustrated in Fig 1B. The premature and thus inactive AFP is 94 amino acids (aa) long and becomes proteolytically trimmed during its passage through the secretory machinery of A. giganteus into the culture supernatant, whereby the secretion leader and an N-terminal prosequence become cut off (10, 11). The sequence of the mature protein contains nine lysines, six tyrosines, and eight cysteines which stabilize the fold by four intramolecular disulfide bonds. The identity of the exact disulfide pairing remains elusive, however, and an isomorphism has been proposed. On the basis of the NMR data obtained in 1995, AFP is thought to adopt four conformations denoted A, B, C, and X (9) (Fig 1C). In this NMR structure, two regions, a cationic loop region (K9, K10, and K32) and a hydrophobic region (Y29, V30, Y45, and Y50), are exposed to the protein surface and have been suggested to be important for intermolecular interactions and recognition. Most importantly, AFP harbors a well-conserved GXC(X_3-9_)C γ-core motif (residues 5 to 14, GKCYKKDNIC; dextromeric isoform) at its N terminus which is part of the cationic loop and conserved across all disulfide bond-stabilized antimicrobial peptides (12). The γ-core motif was recently discovered to be a unifying structural signature present in all cysteine-stabilized antimicrobial peptides of bacterial, fungal, plant, vertebrate, and invertebrate origins (12). It is a conserved three-dimensional structural form existing in three different isoforms, all of which are characterized by two antiparallel β-sheets with an interposed short turn region. Intriguingly, the γ-core motif is present not only in antimicrobial peptides but also in other host defense polypeptides that share membrane interaction as a common mechanism (12, 13). Interestingly, AFP contains a second γ-core motif C-GXC (residues 40 to 49, levomeric isoform 2) at its C terminus. In several studies, the importance of the γ-core motif for antifungal activity of plant defensins was investigated (summarized by Lacerda et al. [14]). Although it was found that the presence or absence of positively charged hydrophobic or neutral amino acids in the γ-core motif determines the antifungal activity, explanations of how this motif mediates the supposed membrane-protein interaction at the molecular level have been elusive.

AFP has been reported to localize at the cell wall and plasma membrane of sensitive filamentous fungi, where it provokes membrane stretching and permeabilization (15) by eventually promoting aggregation of acidic phospholipid vesicles (16). It is furthermore able to bind to chitin and inhibits chitin synthesis in AFP-sensitive filamentous fungi via inhibition of chitin synthase activity (17). Notably, high concentrations of cations inhibit the antifungal activity of the protein (18). In collapsed and dead cells, AFP can also be found intracellularly (15). Still, the (dynamic) mechanisms underlying these observations remain elusive.

In this study, we thus aimed to obtain first insights into the mode of action of AFP at the molecular level by combining computational approaches with wet laboratory experiments. We investigated the predicted A, B, C, and X AFP isoforms by simulating the effect of alterations in the disulfide bridge pairing on the structure of AFP using MD simulations. This approach was complemented with high-performance liquid chromatography (HPLC) and NMR spectroscopy and bioactivity assays of AFP to determine its growth-inhibitory effect on filamentous fungi. In addition to conventional molecular dynamics (cMD) simulations, Gaussian accelerated molecular dynamics (GaMD) simulations (19) were used to model the interaction of AFP with fungal membranes *in silico* beyond the time scales reachable with cMD (19–23).

## RESULTS

### Modeling of AFP in solution.

The NMR structure of the mature 51 aa-long AFP of A. giganteus (PDB: 1AFP) (9) served as the template for generating an initial protein model. In order to investigate the configurational isomerism in the sulfur bridging proposed earlier (9), the four A, B, C, and X models were generated with different sulfur pairings (Fig 1C). All residues were protonated at pH 7.0, and the termini were modeled as charged. This setup led to a total net charge of +9 e^−^ for a single AFP molecule.

**FIG 2:**
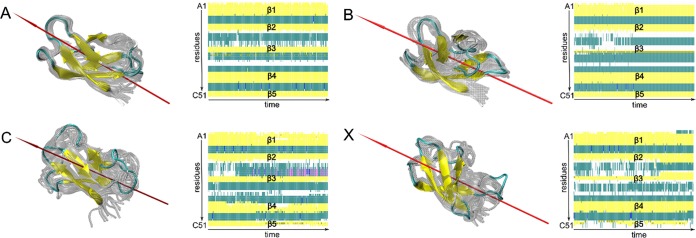
AFP isomers in aqueous solution. The conformational spaces spanned by AFP isomers A, B, C, and X and corresponding secondary evolution plots as predicted by cMD simulations (gray) are shown. Equilibrated structures and dipole moments (red arrow) resulting from the first 300-ns-long MD are highlighted in color. Secondary structure evolution plots from trajectory 1 follow the following color code: yellow for β-sheet region, pink for α-helix region, blue for 3–10 helix region, green for turn region, and white for coil region.

**FIG 3:**
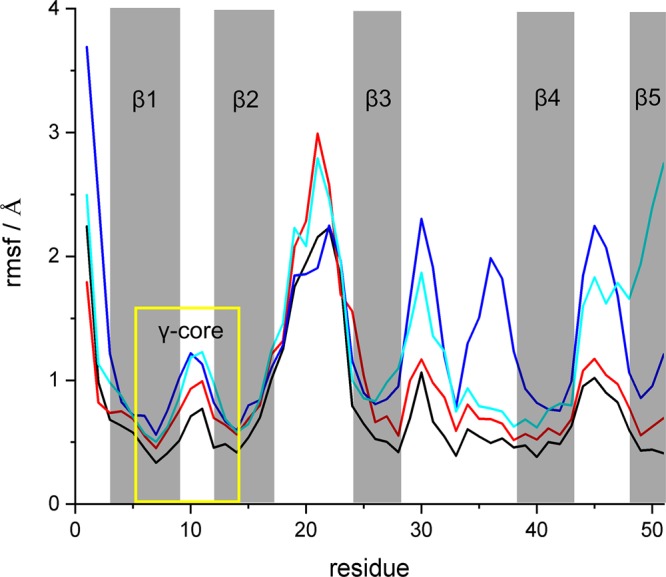
Root mean square fluctuation (rmsf) of the C_α_ atoms in all four isoforms (A, black; B, red; C, blue; X, cyan) in solution averaged over three 300-ns-long cMD trajectories. The five β-strands are highlighted in gray. The γ-core is marked with a yellow box.

Alternations in the sulfur pairing affect the secondary structure and the overall folding of AFP only slightly (Fig. 2). During 300-ns-long cMD simulations, all isoforms conserved the β-barrel shape observed in the NMR structure. However, isomer A showed the lowest deviation with respect to the NMR geometry and the highest structural stability as reflected by the analysis of root-mean-square deviations (RMSD) and fluctuations (RMSF) of the C_α_ atoms (Fig. 3) as well as by secondary structure evolution plots (Fig. 2). In particular, the structural differences between the four isomers were ascribed to the increased flexibility of the protein backbone at loop regions and the terminal ends. While the β-barrel folds remained practically intact, the degree of fluctuation of the loops was strongly dependent on the sulfur pairing pattern (see below).

Isomer A was the most rigid and stayed very close to the proposed favored NMR structure, with a RMSD of less than 1.4 Å (Table 1), since it harbors three (of a maximum of four) cross-links between the β-strands (Fig 1B). Isomers B, C, and X (characterized by only one cross-link between the β-strands) exhibited larger overall distortions in different regions of AFP as indicated by their RMSF values. In particular, isomers B and X, lacking the C26-C49 bridge which connects strands β3 and β5, showed stronger fluctuations in the hydrophobic loop 1 (A18 to T23) located between strands β2 and β3. In isomer C, where the preserved C7-C33 bridge between strands β1 and β3 is missing, high levels of fluctuations in the adjacent loop 2 (C28 to K32 and R35 to A38) were observed. In addition, the largest structural distortions in model X emerged at the terminal ends.

**TABLE 1:** Mean RMSD values (Å) of the protein backbone and dipole moment strengths (debye) of the four AFP isomers computed for three independent 300-ns-long cMD trajectories (standard deviations are given in parentheses)

Model	Trajectory 1	Trajectory 2	Trajectory 3	Mean
RMSD				
A	0.91 (0.15)	1.43 (0.30)	1.35 (0.45)	1.23 (0.40)
B	1.76 (0.23)	2.86 (0.25)	1.75 (0.28)	2.12 (0.58)
C	3.09 (0.87)	2.59 (0.65)	2.22 (0.63)	2.63 (0.81)
X	2.03 (0.49)	2.65 (0.49)	2.17 (0.55)	2.28 (0.58)
Dipole moment				
A	92 (23)	107 (33)	127 (26)	109 (31)
B	78 (23)	87 (28)	84 (30)	83 (27)
C	133 (34)	153 (34)	145 (37)	144 (36)
X	105 (34)	132 (41)	91 (31)	109 (39)

This analysis suggests that model A represents the most likely secondary structure conformation adopted by AFP in aqueous solution. Interestingly, the structure of the N-terminal γ-core motif which partially includes strand β1 and turn 1 was affected by sulfur pairing patterns only marginally, with RMSF values below 1.5 Å, and remained very close to the NMR structure in all models.

In addition to the structural integrity, the electrostatic properties of AFP play an important role in intermolecular contact and recognition. Accordingly, the strength and direction of the dipole moment are good measures for estimating electrostatically driven interactions. During the simulations in aqueous solution, the direction of the dipole moment stayed nearly constant in all models as depicted in Fig. 2. However, the strength of the dipole moment depends on the sulfur pairing pattern (Table 1) as a result of the distinct structural flexibility of the loops and terminal regions of the four isomers. The strongest average dipole moment was found for isomer C (144 debye), with the highest RMSF values at the C termini (Fig. 3), while model B showed the weakest average dipole moment (83 debye). In conclusion, we expect that all four isomers will form similar electrostatically driven contacts with potential interaction partners. However, changes in the dipole moment strength might determine the strength and kinetics of these interactions.

### Isolation of AFP isomers by reversed-phase HPLC.

**FIG 4:**
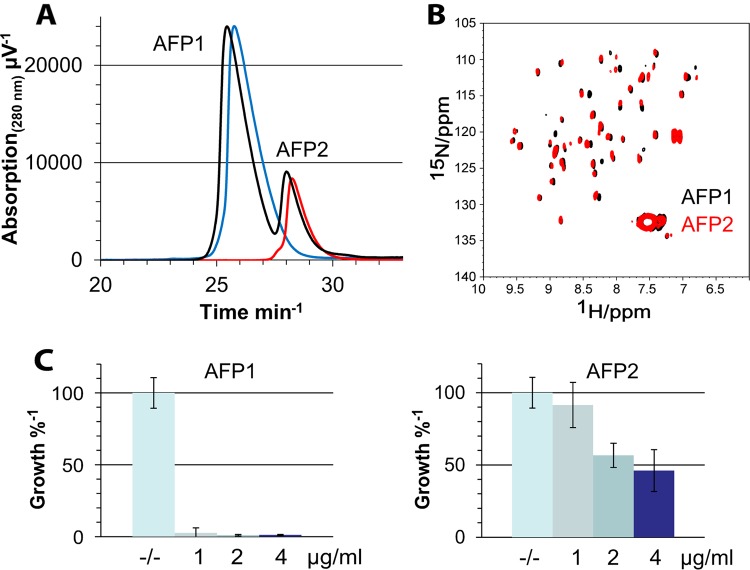
Comparison of AFP variants in HPLC assay, NMR spectroscopy, and bioactivity assay. (A) AFP isolated by cation exchange chromatography eluted in two fractions (black) denoted AFP1 and AFP2. (B) Reinjection of the fractions resulting in lead chromatograms of AFP1 (blue) and AFP2 (red). Both AFP variants were analyzed by NMR spectroscopy. ^1^H,^15^N-SOFAST-HMQC results of experiments exploiting the natural abundance of ^15^N-atoms were recorded and are presented as overlaid data, with AFP1 marked black and AFP2 marked red. (C and D) AFP1 (C) and AFP2 (D) showed different inhibitory efficacies on the fungal test organism A. niger.

Reversed-phase-HPLC was used to separate AFP isomers, if any, from the culture supernatant. Accordingly, two stable AFP variants were identified and named AFP1 and AFP2 (Fig 4A). Both AFP variants were analyzed by NMR spectroscopy, with the results corroborating their monomeric state and suggesting that they differ by only minor structural variations (Fig 4B). Interestingly, bioactivity assays showed that the AFP1 variant exhibits antifungal activity against A. niger that is more than 4 times higher than that exhibited by AFP2 (Fig 4C). The differences between AFP1 and AFP2, however, did not result from different disulfide bridge pairings but from modifications in the amino acid sequence. Mass spectroscopy (MS) analysis disclosed that the less-active form, AFP2, lacks the first N-terminal residue (alanine) of the mature protein. This is the first time that a truncated AFP variant has been observed and might suggest that proteolytic maturation of AFP during its passage through the secretory pathway of A. giganteus could be error-prone.

### AFP-membrane interaction.

Fungal membranes differ from other eukaryotic membranes by an increased fraction of sphingolipids (20% to 30%) (24) and ergosterol (25). Furthermore, membranes of filamentous fungi contain special acidic glycosylphosphatidylinositols (GPIs), namely, glycoinositol phosphoryl ceramides (GIPCs), which are not synthesized in mammalian cells (26). Details of the composition of the complete *in silico*-constructed bilayer membrane are given in Table 2. Overall, the bilayer was highly anionic and carried a negative net charge of 148 e^−^.

**TABLE 2:** Composition of the lipid model membrane (24)

Lipid(s)	Abbreviation	Total (per leaflet)	Charge/lipid
Acidic glycosphingolipids	GIPC	92 (46)	−1
Ergosterol	Ergosterol	132 (66)	0
Saturated phospholipids			
Phosphatidylcholine	DPPC (16:0)	28 (14)	0
Phosphatidylethanolamine	DPPE (16:0)	28 (14)	0
Phosphatidylglycerol	DPPG (16:0)	28 (14)	−1
Unsaturated phospholipids			
Phosphatidylcholine	DUPC (18:2)	28 (14)	0
Phosphatidylethanolamine	SLPE (18:2)	28 (14)	0
Phosphatidylglycerol	SLPG (18:2)	28 (14)	−1

**FIG 5:**
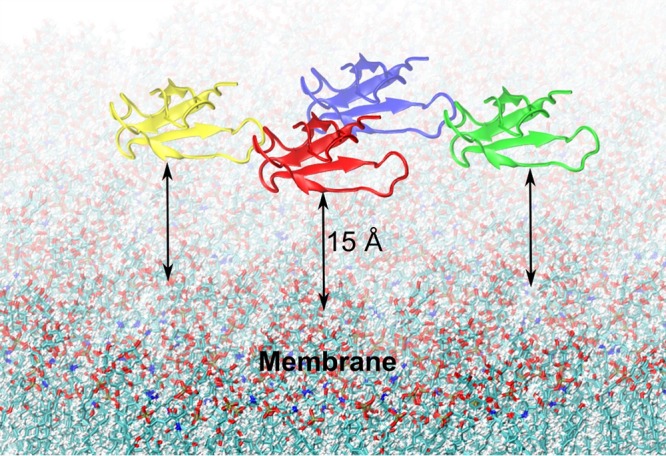
Initial configuration of AFP on a fungal model membrane. Four AFP molecules were placed in aqueous solution at a distance of approximately 15 Å from the fungal membrane surface.

In order to model the interaction between AFP and fungal membranes, four AFP molecules (denoted I, II, III, and, IV) were placed in aqueous solution at a distance of about 15 Å above the membrane surface (Fig. 5). Such a setup, on the one hand, allows investigation of interpeptide interactions which may eventually lead to aggregation and, on the other hand, improves the statistical analysis by sampling the conformational space of individual molecules and their interactions with the membrane. All four AFP molecules were configured according to sulfur-pairing model A, which showed the highest stability in the protein-solvent simulations (see above) and the best agreement with the NMR structure. The resulting negative charge of the protein-membrane system was neutralized by adding sodium counterions to the aqueous TIP3P (27) solution.

**FIG 6:**
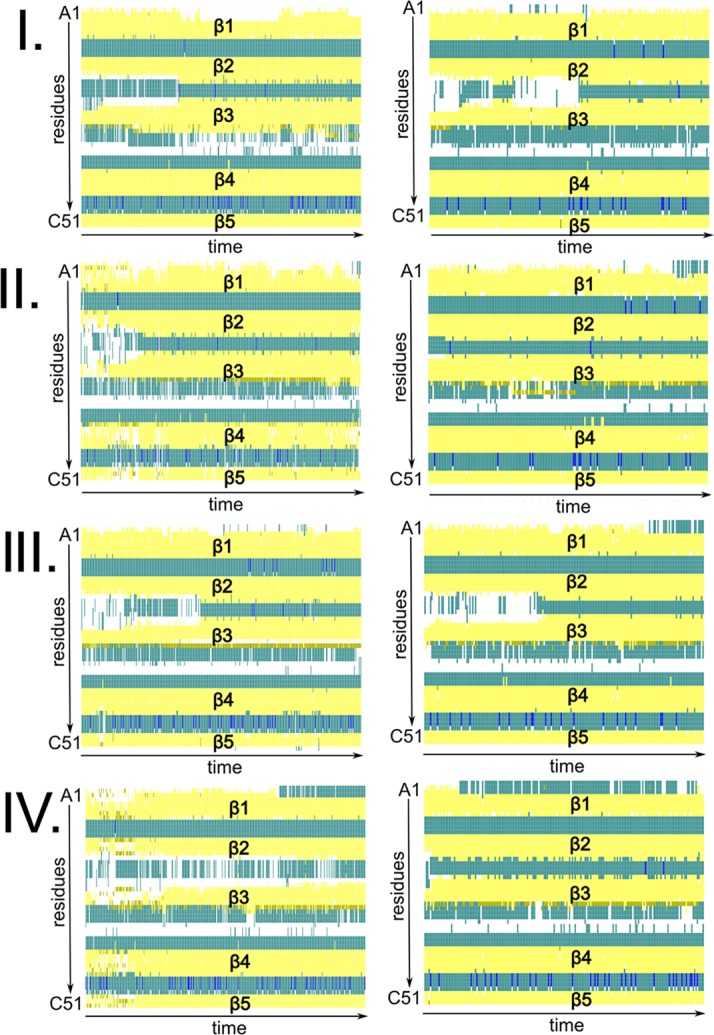
Secondary structure evolution plot of four AFPs (configured according to isoform A) denoted I, II, III, and IV at the membrane interface in cMD (left) simulations and GaMD (right) simulations. Yellow, pink, blue, green, and white indicate β-sheet, α-helical, 3–10 helical, turn, and coil regions, respectively.

Both simulation techniques (cMD and GaMD) resulted in conserved protein secondary structures at the membrane interface as observed for AFP in solution (Fig. 6). The average RMSD values determined for the protein backbone were slightly higher than in solution but did not exceed 3 Å.

**FIG 7:**
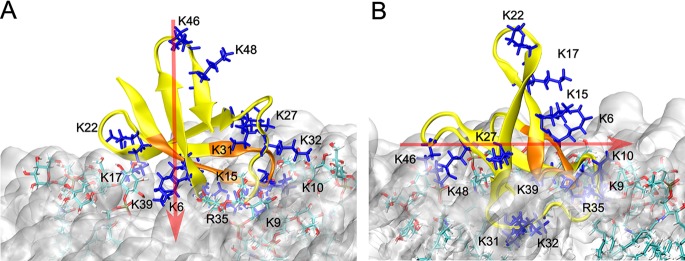
Interaction of AFP in conf1 (A) and conf2 (B) with fungal membrane. The protein and its N-terminal γ-core are highlighted in yellow and orange, respectively. All lysine and arginine residues are depicted as blue sticks, while GIPCs interacting with AFP are shown as colored in licorice representations. The bilayer membrane is drawn as a white cloud. The dipole moments of AFP are depicted by the red arrows.

All four AFP molecules strongly attached to the membrane surface without penetrating the hydrophobic core of the bilayer. Examples are shown in Fig. 7. The adsorption was mainly driven by electrostatic attraction between the positively charged protein and the acidic membrane surface containing GIPC and phosphatidylglycerol (PG) lipids. The sugar and phosphate groups of GIPCs played a crucial role in the attachment of AFP on the hydrophilic membrane surface. Due to their size, GIPC lipids emerge from the membrane into the aqueous solution and, consequently, form hydrogen bonds and salt bridges with AFP.

After the initial attachment and subsequent reorientation of the peptides on the membrane surface, all AFP molecules in GaMD and two of four AFPs in cMD simulations adopted configuration -conf1- (Fig 7A), which was mostly maintained during the course of the simulations. In this configuration, the dipole moments of the individual AFP molecules were aligned to the strong electric field of the membrane and pointed toward the bilayer. The orientation of the dipole moment within the structure of AFP did not significantly change in comparison to simulations performed in aqueous solution. Its strength, however, nearly doubled under the influence of the electric field of the bilayer. Notably, the dipole moment strength and its fluctuation in the GaMD simulation were on average slightly increased compared to the cMD simulation data (Table 3).

**TABLE 3:** Average dipole moments of individual AFP molecules and average interaction energies between AFP and membrane^*a*^

Molecule	Dipole moments (debye)		AFP-membrane interaction energy (kcal/mol)	
	cMD	GaMD	cMD	GaMD
I	148 ± 24	169 ± 25	−1,414 ± 132	−1,057 ± 271
II	133 ± 27	207 ± 32	−1,143 ± 134	−1,617 ± 235
III	198 ± 20	221 ± 28	−816 ± 220	−1,901 ± 167
IV	208 ± 29	229 ± 31	−1,441 ± 197	−2,011 ± 200

a Averages were computed over the last 100 ns in cMD and GaMD simulations at the membrane. Energies were estimated with the NAMDEnergy plugin of VMD (52).

In the GaMD simulation, lysine residues of strand β1 (K6) of the N-terminal dextromeric γ-core motif and β2 (K15 and K17) which are spatially adjacent to the γ-core motif strongly contributed to the membrane attachment by forming stable salt bridges and hydrogen bonds with the negatively membrane components. Moreover, K9 and K10 (located in the γ-core) and K39 of strand β4 exhibited additional interactions. The other strands stayed mostly away from the bilayer; also, the C-terminal levomeric γ-core motif did not participate in membrane interaction. Besides the strands, two turns, turn 2 (K22) and turn 3 (R35), contributed to the membrane attachment. Strong electrostatic interactions between AFP and the membrane were also predicted in the GaMD simulation as indicated by favorable interaction energy values of about -2,000 kcal/mol (Table 3). The unfavorable interaction energy and large standard deviation observed for AFP I reflect an unpropitious orientation and high flexibility of this molecule, which were not predicted for the other AFP molecules. Other lysine residues, such as K27 and K32, established short-living interactions with the membrane and led only to transient reorientations, indicating energetically less favorable orientations such as were observed for molecule I.

While GaMD simulations yielded a uniform orientation (conf1) of all four AFP molecules on the membrane surface, only two AFP molecules (I and IV) adopted this configuration in cMD simulations. The other two AFP molecules (II and III) behaved differently at the bilayer interface and were trapped in another orientation (conf2) (Fig. 7B). The conformational discrepancies between the individual AFP molecules in cMD simulations are reflected by the minimum distances between membrane atoms and selected AFP residues (Table 4). In agreement with the GaMD findings, AFP molecules I and IV interact with the membrane via their β-strands (β-strands 1 and 2), including K6, K15, and K17, which lie on average about 3 Å away from the membrane oxygen atoms. In contrast, AFP II and AFP III molecules adopt the conformation conf2 where the three lysines basically do not interact with the membrane. Instead, the interaction with the membrane is established through turn 3, including K31, K32, and R35. In addition to these key residues, strand β4 (R35 and K39) and the γ-core (K9) also contributed to surface attachment in both orientations.

**TABLE 4:** Averaged distances (Å) between amino/guanidine nitrogen molecules of lysine and arginine residues of AFP molecules and oxygen atoms on the membrane^*a*^

Residue	I	II	III	IV
K6	2.97± 0.28 (3.85 ± 1.46)	10.41 ± 2.51 (3.17 ±0.57)	3.64 ± 0.88 (2.83 ± 0.08)	3.04 ± 0.46 (3.19 ± 0.59)
K9	3.22 ±0.78 (3.10 ± 0.56)	3.2 ± 0.65 (4.14 ± 1.13)	3.76 ± 1.01 (3.59 ± 0.94)	3.52 ± 0.90 (3.89 ± 1.30)
K10	5.09 ± 1.69 (3.73 ± 0.98)	3.53 ± 0.93 (4.16 ± 1.24)	4.48 ± 1.90 (4.02 ± 1.41)	5.29 ± 2.36 (3.54 ± 1.03)
K15	3.77 ± 0.96 (5.06 ± 1.92)	12.45 ± 1.52 (2.99 ± 0.37)	7.87 ± 2.85 (3.20 ± 0.44)	4.30 ± 1.52 (3.31 ± 0.73)
K17	3.13 ± 0.40 (3.91 ± 1.25)	17.82 ± 2.66 (2.86 ± 0.11)	7.09 ± 2.44 (2.82 ± 0.09)	3.16 ± 0.65 (3.03 ± 0.12)
K22	3.43 ± 0.98 (6.00 ± 2.73)	22.95 ± 3.09 (3.17 ± 0.63)	16.60 ± 3.24 (4.50 ± 1.23)	3.52 ± 0.98 (3.37 ± 0.83)
K27	13.73 ± 1.78 (8.46 ± 1.90)	3.30 ± 0.72 (6.16 ± 1.65)	13.86 ± 2.31 (10.75 ±1.75)	14.59 ± 1.93 (7.41 ± 1.95)
K31	8.26 ± 2.12 (10.23 ± 3.54)	3.77 ± 1.39 (10.95 ± 2.79)	4.22 ± 1.88 (10.19 ± 2.07)	5.39 ± 1.78 (12.08 ± 2.62)
K32	9.09 ± 2.57 (5.89 ± 1.99)	3.12 ± 0.45 (9.54 ± 2.71)	4.03 ± 1.69 (9.43 ± 2.40)	8.55 ± 2.54 (10.23 ± 3.07)
R35	2.84 ± 0.08 (3.30 ± 0.54)	3.51 ± 0.73 (3.44 ± 0.73)	2.93 ± 0.17 (2.93 ± 0.22)	3.44 ± 0.63 (3.13 ± 0.44)
K39	2.97 ± 0.35 (7.02 ± 2.51)	4.92 ± 2.11 (3.17 ± 0.57)	2.89 ± 0.09 (2.83 ± 0.08)	3.04 ± 0.43 (3.19 ± 0.59)
K46	16.88 ± 2.96 (22.51 ± 2.94)	4.01 ± 1.29 (20.47 ± 1.45)	10.85 ± 2.36 (19.11 ± 2.36)	14.11 ± 2.27 (20.01 ± 2.05)
K48	19.29 ±1.22 (18.95 ± 1.62)	3.76 ± 0.91 (12.67 ± 1.54)	13.68 ± 1.86 (16.83 ± 1.45)	15.84 ± 1.03 (14.74 ± 3.61)

a Average values were computed over the last 100 ns of cMD simulations predicted by cMD and GaMD (in parenthesis).

**FIG 8:**
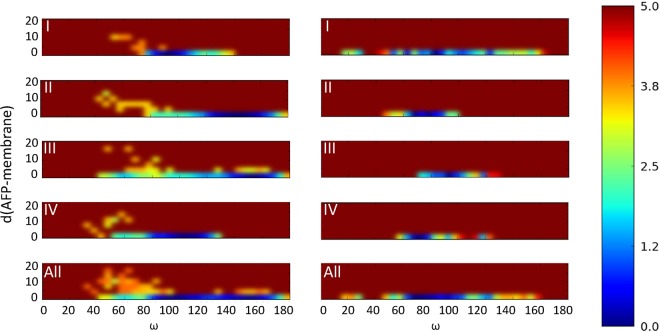
Two-dimensional (2D) potential of mean force (PMF) in cMD (left) and GaMD (right) simulations. 2D potential of mean force (PMF) describes the change of free energy as a function of the protein orientation defined by the ω angle between the membrane normal and a vector determined by the C_α_ atoms of K22 and K39 of AFP and the minimum distance to the membrane. The PMF profiles of the individual AFP molecules (I to IV) and the combined profile (All) were computed from 400-ns cMD simulations and from 400-ns GaMD, simulations applying cumulant expansion of the second order. Energy scale data are indicated in kilocalories per mole.

The two configurations can be characterized by the angle between the axis defined by the C_α_ atoms of K22 and K39 of AFP and the membrane normal. While angles between 70° and 100° are ascribed to conf1, conf2 is characterized by angles between 140° and 160°. These findings suggest that there are at least two stable orientations which the AFP protein can adopt on the bilayer in cMD simulations. The optimal orientation of AFP on the membrane can be analyzed through the potential of mean force (PMF) profiles (Fig. 8). The reweighted profile of the GaMD simulation using the cumulant expansion of the second-order approach (28) identified conf1 as energetically favorable in comparison to conf2. Although data points were collected only every 0.2 ns and the system suffered a high noise level, the trend is clearly visible. The free-energy profiles also show that the membrane interaction occurred much faster in the GaMD simulations than in the cMD simulations since the densities of nonadsorbed AFP are visible only in the cMD PMF profiles.

Despite the presence of four AFP units in the simulation cell, we never observed any intermolecular interaction between AFP molecules. Thus, dimerization or oligomerization of AFP molecules in solution in the presence of a lipid bilayer is unlikely. The lack of any intermolecular AFP interactions can be rationalized as being the result of electrostatic repulsion between the strongly positively charged proteins. This observation is in full agreement with the NMR data described above.

The dynamical behavior of lipids in membranes may be significantly altered upon interaction with adsorbates, such as AFP molecules. In order to investigate this issue, lateral diffusion coefficients of all lipids on upper and lower leaflets of the fungal membrane model were estimated from the MD simulations (Table 5). These values range from 7 × 10^−8^ cm^2^/s to 11 × 10^−8^ cm^2^/s and are in good agreement with lateral diffusion coefficients predicted and measured for lipids in the past (22, 29).

**TABLE 5:** Lateral diffusion coefficients (*D*) of lipids of the upper and lower leaflet of the model fungal membrane calculated from cMD and GaMD simulations (diffusion coefficients from GaMD are in parentheses)

Lipid	*D*_upper_ (10^-8^ cm^2^/s)	*D*_lower_ (10^-8^ cm^2^/s)	*D*_upper_ – *D*_lower_ (10^-8^ cm^2^/s)
Acidic glycosphingolipids (GIPC)	7.9 (6.9)	8.6 (8.9)	−0.7 (−2.0)
Dipalmitoylphosphatidylcholine (DPPC)	8.5 (8.9)	8.8 (11.1)	−0.3 (−2.2)
Dipalmitoylphosphatidylethanoamine (DPPE)	8.5 (8.3)	8.9 (7.7)	−0.4 (0.6)
Dipalmitoylphosphatidylglycerol (DPPG)	8.5 (9.1)	9.7 (9.3)	−1.2 (−0.2)
Unsaturated dipalmitoylphosphatidylcholine (DUPC)	8.2 (8.2)	9.6 (11.0)	−1.4 (−2.8)
Ergosterol (ERG)	8.3 (7.9)	9.1 (10.5)	−0.8 (−2.6)
Unsaturated phosphatidylethanoamine (SLPE)	8.9 (10.1)	9.1 (8.1)	−0.2 (2.0)
Unsaturated phosphatidylglycerol (SLPG)	8.3 (7.6)	9.2 (11.2)	−0.9 (−3.6)

According to our simulations, the mobility of nearly all lipids of the upper leaflet of the fungal membrane was drastically reduced upon AFP binding compared to that seen with the “AFP-free” lower leaflet as reflected by the negative sign of the *D*_upper_ − *D*_lower_ values. These findings are qualitatively reproduced by the two simulation techniques and suggest that AFP alters local membrane fluidity.

## DISCUSSION

In this study, we demonstrated that different isoforms of AFP due to cysteine shuffling as previously reported (9) do not exist. The interpretation of NMR spectra from 1995 was probably a result of improper purification of the AFP sample, which was done only by cation exchange chromatography and desalting. It is very likely that the authors measured a mixture of both of the AFP variants (AFP1 and AFP2) which we have identified in the current study. Also, all previous structural information was collected from homonuclear spectra, which are less well resolved and show more overlapping signals than spectra from heteronuclear experiments. On the basis of the reversed-phase HPLC and NMR spectra of AFP1 and AFP2, we conclude that both variants have only one cysteine conformation, which most likely corresponds to model A. The remaining models (B, C, and X) are less probable due to the presence of larger overall distortions during MD simulations. Although MD simulations in aqueous solution showed a stable secondary structure of all disulfide bridge isomers, only model A was structurally very close to the NMR structure determined in 1995 (9). Therefore, subsequent cMD and GaMD simulations at the membrane interface were performed only with model A.

According to the simulations, unfolding of AFP upon membrane interaction, (subsequent) membrane integration, and direct permeabilization of the fungal membrane are unlikely events. Also, we consider the formation of membrane pores consisting of multiple AFPs to be improbable, as the simulations do not suggest protein-protein interactions due to strong electrostatic repulsions. Instead, both the cMD and GaMD simulations clearly suggested that AFP builds a well-ordered carpet on the outer leaflet of the lipid bilayer, in contrast to MD simulations of other peptides, for which insertion is computationally predicted (30, 31).

Notably, AFP configured according to model A adopts a well-defined orientation in its membrane-bound state in which the conserved N-terminal γ-core motif directly faces the bilayer and the C-terminal γ-core motif points away from the membrane. This orientation is induced by electrostatic attraction mediated by residues of the cationic region (K9 and K10 but not K32), which is strongly enhanced at anionic membranes.

Furthermore, simulations in aqueous solution demonstrated that, independently of the disulfide pairing and even after removal of the preserved disulfide bridge between strands β2 and β3, the γ-core motif remained structurally very stable, in agreement with calorimetric measurements (16). Interestingly, the C-terminal γ-core motif had no impact on membrane adsorption, which might offer an explanation of why it is not conserved in the other 50 members of the AFP family (8). Similarly, and in contrast to recent expectations, residues of the hydrophobic region (Y29, V30, Y45, and Y50) were also not found to be involved in AFP-membrane interactions. This suggests that the hydrophobic patch might play a role at later stages of the AFP mechanism but not during its initial attachment to the membrane.

The N terminus of AFP seems to be dispensable for the protein-membrane interaction since it does not participate in the interaction site. As AFP1 and AFP2 differ in both their N termini (AFP2 lacks alanine at position 1) and their antifungal activities, it is likely that the AFP moiety opposite the membrane interaction site plays a role in antifungal activity. It is tempting to speculate that this moiety might interact with chitin and/or chitin synthases bound to the membrane, a process which cannot be modeled yet with all-atom MD simulations.

In particular, MD simulations predicted that AFP alters membrane fluidity. Since membrane fluidity is crucial for the fungal defense against antifungal agents (32), any inhibition thereof would amplify the growth-inhibitory effect of AFP.

In general, the data collected from the cMD and GaMD simulations showed mutual corroboration. However, while the cMD simulations suggested at least two (stable) orientations (conf1 and conf2) of AFP on the membrane, GaMD simulations predicted conf1 as most likely. This discrepancy can be explained in terms of the thermodynamic stability of these orientations. Although we do not have a direct proof for this hypothesis, it seems reasonable that conf2 is energetically less favored as indicated by the calculated PMF profiles. While the large energy barrier separating conf1 and conf2 in the cMD simulations could not be traversed within the nanosecond time scale, application of a boost potential in the GaMD simulations smoothed the energy landscape, allowing transitions between the two states. Since the energy of conf1 is lower than that of conf2, AFP prefers staying in conf1 and seldom adopts conf2. This means that unbiased cMD simulations on the nanosecond time scale result in insufficient sampling for describing equilibrium conditions of the AFP-membrane system. Nevertheless, both the cMD and GaMD simulations identified the γ-core motif as an “interaction hot spot” with the membrane, which, to the best of our knowledge, was demonstrated here for the first time.

### Conclusion.

In this work, we validated the stability of the β-barrel fold of AFP and suggested that model A is the most probable and only naturally occurring isoform. This was confirmed by NMR and MS analyses. Furthermore, we demonstrated that AFP strongly interacts with an anionic fungal membrane model without penetrating into its hydrophobic inner part. The γ-core strongly contributes to membrane binding without significantly losing its structure. This seems not to be the case for the N terminus of AFP, which is most likely involved in a latter step of fungicidal mechanism, as reflected by the bioactivity assays of the AFP1 and AFP2 variants. Summarizing, we used cMD and GaMD simulations, backed by experimental evidence, as an elegant way to identify important features of AFP and the dynamics of its membrane interaction. The hypotheses generated in this work can now be experimentally verified by targeted protein modifications in future experiments.

## MATERIALS AND METHODS

### MD simulations in solution.

All MD simulations of AFP in Cl^−^−neutralized aqueous TIP3P solution (27) were performed with the NAMD2.10 CUDA version (33) using the CHARMM27 (CHARMM22 + CMAP) force field (34). After energy minimization performed with the conjugated gradient algorithm, the systems were heated to 300 K and thermally equilibrated for 20 ps. The subsequent 300-ns-long production runs were performed under constant conditions with respect to the number of particles (N), pressure (P), and temperature (T). This isothermal-isobaric (NPT) ensemble at atmospheric conditions (T = 300 K and P  = 1 atm) was realized by the Langevin piston algorithm (35). Short-range electrostatics and van der Waals interactions were truncated above 12 Å. Long-range electrostatic interactions in the periodic system were calculated using the Particle Mesh Ewald summation (36). The time step of 2 fs was enabled by the SHAKE algorithm (37), freezing all bonds containing hydrogen atoms. All simulations were repeated three times. In total, 12 300-ns-long MD simulations were performed.

### MD simulations at the membrane interface.

Interaction of AFP configured according to model A with the fungal membrane was simulated with two different techniques, namely, cMD and GaMD (19). The bilayer membrane was built based on the available knowledge for fungal membrane compositions with the CHARMM-GUI (38, 39) program using the CHARMM36 force field for lipids (40, 41) and carbohydrates (42). Modifications used for acidic GIPCs were added with homemade patches.

The AFP-membrane model consisted of four AFP molecules on a 108-by-108-Å^2^ membrane, which provides better statistics, as the dynamics of each molecule can be evaluated separately.

The 400-ns-long isothermal-isobaric cMD simulations with the Langevin piston method (35) were preceded by energy minimization and thermal equilibration at 300 K. Bonds containing hydrogen atoms were restrained by the SHAKE algorithm (37), allowing a time step of 2 fs. Van der Waals interactions were cut at 12 Å, while coulomb interactions were calculated using the Particle Mesh Ewald summation (36). Fluctuations in the cell size were allowed only in perpendicular orientation to the membrane plane, keeping the membrane density unchanged during the simulation. cMD simulations were performed with the CPU version of NAMD2.10.

Accelerated MD simulations flatten the potential energy landscape to accelerate biomolecular transitions between energy minima (20). In the GaMD variant, the applied boost potential follows a Gaussian distribution which allows accurate reweighting for determining thermodynamic observables (19). GaMD calculations were performed with the CUDA version of Amber14 (43) using the CHARMM36 force field as described above and an average acceleration of 204 kcal/mol. The standard routine of the dual-boost acceleration was applied for flattening the total potential energy of the system and the energy landscapes of all dihedrals. After energy minimization and thermal equilibration, the system was subjected to a 400-ns-long production run in an isothermal-isobaric ensemble with fixed cell size along the membrane plane and under periodic boundary conditions. This was realized by Langevin dynamics (44) using a pressure relaxation time τ*_P_* = 2.0 ps and a collision frequency γ = 2.0 ps^−1^. Electrostatic and van der Waals interactions were truncated above 8.0 Å. Beyond this cutoff, electrostatic interactions were treated with the Particle Mesh Ewald summation (36). A 2-fs time step was allowed by applying SHAKE method (37) to bonds involving hydrogen.

### Lateral diffusion coefficient of lipids.

The lateral diffusion coefficients of all lipids were computed according to the Einstein relation (equation 1) (22) as follows:

$$D=\frac{1}{N}\overset{N}{\underset{i}{\sum }}\frac{1}{4t}{\left|r_{i}\left(t\right)-r_{i}\left(0\right)\right|}^{2}$$

where *N* represents the number of lipids and *r*_i_ the coordinates of the center of mass of each lipid. Time window *t* between two points at time zero and time *t* was set to 10 ns. During the last 300 ns, the time window was shifted along the trajectory in 1-ns time steps to get averages for the lipid diffusion. The first 100 ns were skipped since the membranes were not equilibrated.

### AFP production and purification.

AFP was purified from batch cultures of A. giganteus strain IfGB15/0902 cultivated in stirred tank bioreactors under controlled conditions (BioStat; Sartorius) (4-liter working volume). After inoculation (10^6^ spores/ml) of complete medium, consisting of minimal medium (45) supplemented with 1% yeast extract and 0.5% Casamino Acids, cultivation was performed at 28°C for 96 h. Secreted AFP was isolated from culture supernatants as well as from biomass. Biomass and culture broth were separated by filtration. AFP isolation from biomass was achieved by incubating the cell suspension in 10 mM Tris (pH 7)–1 mM EDTA–1.5 M NaCl for 2 h. AFP isolation from the culture broth was performed via chitin chromatography using crab shell chitin (Fluka). After 2 h of incubation, chitin was washed with 10 mM Tris (pH 7)–1 mM EDTA before AFP was eluted with 10 mM Tris (pH 7)–1 mM EDTA–1.5 M NaCl. AFP extracted from both sources was applied to cation exchange chromatography, after which the protein was eluted in single-peak purity. All fractions containing AFP were pooled, and the solvent was exchanged to 0.01% trifluoric acid by dialysis. A final quality control of the AFP sample purity was done by reverse-phase HPLC, corroborating more than 95% purity.

### NMR and MS analysis.

During reverse-phase HPLC, we could distinguish and separate two forms of the AFP, denoted AFP1 and AFP2, which were analyzed and characterized by mass spectrometry (MS) and nuclear magnetic resonance (NMR). Samples for NMR spectroscopy were prepared by dissolving 2.8 mg (AFP1) and 1.4 mg (AFP2) of protein, received by HPLC fraction collection, in 500 µl H_2_O with 0.01% trifluoroacetic acid (TFA). The solution was transferred to a 5-mm sample tube, and a capillary filled with D_2_O was added. NMR spectra were recorded at 300 K and 750 MHz (^1^H frequency) on Bruker AV-III spectrometers using a cryogenically cooled 5-mm-long TCI-triple resonance probe equipped with one-axis self-shielded gradients and using topspin 3.5pl7 (Bruker) as the acquisition software. One-dimensional spectra and total correlation spectroscopy (TOCSY) (46, 47) and nuclear Overhauser effect spectroscopy (NOESY) (48) data were recorded using 2,048-by-256 complex data points, 16 scans for AFP1, and 32 scans for AFP2. Water suppression was achieved using WaterGATE (49, 50). In addition, a ^1^H,^15^N-SOFAST-HMQC (45) was recorded using 512-by-256 complex data points and 1,536 and 6,144 scans for AFP1 and AFP2, respectively. Data were processed using topspin 3.5pl7, and data matrices were 4,096-by-2,048 real data points; a squared cosine shifted by 90° was used as window function.

Both AFP variants were also investigated by electrospray ionization (ESI) mass spectrometry. Measurements were performed on an Orbitrap Elite mass spectrometer (Thermo Scientific). The AFP proteins were dissolved in 0.01% formic acid (1.5 µg/µl) and directly injected using a flow rate of 4 µl/min. Mass spectra from *m*/*z* of 200 to 2,000 were acquired with a nominal resolution of 240,000 and an AGC target value of 1e6. Scans were averaged and deconvoluted using the Xtract method of program deconvolution 3.0 software (Thermo Scientific).

### AFP bioactivity assays.

The growth-inhibitory effect of AFP on the test organism A. niger was determined using an in-microtiter plate standard protocol as described before (51). In brief, 10^3^ spores of A. niger were used to inoculate 150 μl yeast extract-peptone medium in the absence or presence of different AFP concentrations (1 to 4 μg/ml). The cultures were inoculated at 28°C for 28 h at 120 rpm. Spore germination and growth were assessed by measuring the optical density at 600 nm of biological replicates (quintuplicates).

## References

[1] Enoch DA, Ludlam HA, Brown NM 2006 Invasive fungal infections: a review of epidemiology and management options. J Med Microbiol 55:809–818. doi:10.1099/jmm.0.46548-0. 16772406

[2] Perlin DS, Shor E, Zhao Y 2015 Update on antifungal drug resistance. Curr Clin Microbiol Rep 2:84–95. doi:10.1007/s40588-015-0015-1. 26120512 PMC4479306

[3] Meyer V, Andersen MR, Brakhage AA, Braus GH, Caddick MX, Cairns TC, de Vries RP, Haarmann T, Hansen K, Hertz-Fowler C, Krappmann S, Mortensen UH, Peñalva MA, Ram AFJ, Head RM 2016 Current challenges of research on filamentous fungi in relation to human welfare and a sustainable bio-economy: a white paper. Fungal Biol Biotechnol 3:6. doi:10.1186/s40694-016-0024-8. 28955465 PMC5611618

[4] Pfaller MA 2012 Antifungal drug resistance: mechanisms, epidemiology, and consequences for treatment. Am J Med 125:S3–S13. doi:10.1016/j.amjmed.2011.11.001. 22196207

[5] Kanafani ZA, Perfect JR 2008 Antimicrobial resistance: resistance to antifungal agents: mechanisms and clinical impact. Clin Infect Dis 46:120–128. doi:10.1086/524071. 18171227

[6] Meyer V 2008 A small protein that fights fungi: AFP as a new promising antifungal agent of biotechnological value. Appl Microbiol Biotechnol 78:17–28. doi:10.1007/s00253-007-1291-3. 18066545

[7] Szappanos H, Szigeti GP, Pál B, Rusznák Z, Szűcs G, Rajnavölgyi É, Balla J, Balla G, Nagy E, Leiter É, Pócsi I, Hagen S, Meyer V, Csernoch L 2006 The antifungal protein AFP secreted by Aspergillus giganteus does not cause detrimental effects on certain mammalian cells. Peptides 27:1717–1725. doi:10.1016/j.peptides.2006.01.009. 16500727

[8] Paege N, Jung S, Schäpe P, Müller-Hagen D, Ouedraogo JP, Heiderich C, Jedamzick J, Nitsche BM, Van Den Hondel CA, Ram AF, Meyer V 2016 A transcriptome meta-analysis proposes novel biological roles for the antifungal protein anafp in aspergillus Niger. PLoS One 11:e0165755. doi:10.1371/journal.pone.0165755. 27835655 PMC5106034

[9] Campos-Olivas R, Bruix M, Santoro J, Lacadena J, Martinez del Pozo A, Gavilanes JG, Rico M 1995 NMR solution structure of the antifungal protein from Aspergillus giganteus: evidence for cysteine pairing isomerism. Biochemistry 34:3009–3021. doi:10.1021/bi00009a032. 7893713

[10] Wnendt S, Ulbrich N, Stahl U 1994 Molecular cloning, sequence analysis and expression of the gene encoding an antifungal-protein from Aspergillus giganteus. Curr Genet 25:519–523. doi:10.1007/BF00351672. 8082203

[11] Martínez-Ruiz A, Martínez del Pozo A, Lacadena J, Mancheño JM, Oñaderra M, Gavilanes JG, 1997 Characterization of a natural larger form of the antifungal protein (AFP) from Aspergillus giganteus. Biochim Biophys Acta 1340:81–87. doi:10.1016/S0167-4838(97)00038-1. 9217017

[12] Yount NY, Yeaman MR 2004 Multidimensional signatures in antimicrobial peptides. Proc Natl Acad Sci U S A 101:7363–7368. doi:10.1073/pnas.0401567101. 15118082 PMC409924

[13] Yeaman MR, Yount NY 2007 Unifying themes in host defence effector polypeptides. Nat Rev Microbiol 5:727–740. doi:10.1038/nrmicro1744. 17703227

[14] Lacerda AF, Vasconcelos EAR, Pelegrini PB, Grossi de Sa MF 2014 Antifungal defensins and their role in plant defense. Front Microbiol 5:116. doi:10.3389/fmicb.2014.00116. 24765086 PMC3980092

[15] Theis T, Stahl U 2004 Antifungal proteins: targets, mechanisms and prospective applications. Cell Mol Life Sci C 61:437–455. doi:10.1007/s00018-003-3231-4. PMC11146029 14999404

[16] Lacadena J, Martínez del Pozo A, Gasset M, Patiño B, Campos-Olivas R, Vázquez C, Martínez-Ruiz A, Mancheño JM, Oñaderra M, Gavilanes JG 1995 Characterization of the antifungal protein secreted by mould Aspergillus giganteus. Arch Biochem Biophys 324:273–281. doi:10.1006/abbi.1995.0040. 8554319

[17] Hagen S, Marx F, Ram AF, Meyer V 2007 The antifungal protein AFP from Aspergillus giganteus inhibits chitin synthesis in sensitive fungi. Appl Environ Microbiol 73:2128–2134. doi:10.1128/AEM.02497-06. 17277210 PMC1855660

[18] Theis T, Wedde M, Meyer V, Stahl U 2003 The antifungal protein from Aspergillus giganteus causes membrane permeabilization. Antimicrob Agents Chemother 47:588–593. doi:10.1128/AAC.47.2.588-593.2003. 12543664 PMC151754

[19] Miao Y, Feher VA, McCammon JA 2015 Gaussian accelerated molecular dynamics: unconstrained enhanced sampling and free energy calculation. J Chem Theory Comput 11:3584–3595. doi:10.1021/acs.jctc.5b00436. 26300708 PMC4535365

[20] Hamelberg D, Mongan J, McCammon JA 2004 Accelerated molecular dynamics: a promising and efficient simulation method for biomolecules. J Chem Phys 120:11919–11929. doi:10.1063/1.1755656. 15268227

[21] Miao Y, Feixas F, Eun C, McCammon JA 2015 Accelerated molecular dynamics simulations of protein folding. J Comput Chem 36:1536–1549. doi:10.1002/jcc.23964. 26096263 PMC4487363

[22] Wang Y, Markwick PRL, De Oliveira CAF, McCammon JA 2011 Enhanced lipid diffusion and mixing in accelerated molecular dynamics. J Chem Theory Comput 7:3199–3207. doi:10.1021/ct200430c. 22003320 PMC3191728

[23] Miao Y, Caliman AD, McCammon JA 2015 Allosteric effects of sodium ion binding on activation of the M3 muscarinic G-protein-coupled receptor. Biophys J 108:1796–1806. doi:10.1016/j.bpj.2015.03.003. 25863070 PMC4390834

[24] Tagliari L, Toledo MS, Lacerda TG, Suzuki E, Straus AH, Takahashi HK 2012 Membrane microdomain components of Histoplasma capsulatum yeast forms, and their role in alveolar macrophage infectivity. Biochim Biophys Acta 1818:458–466. doi:10.1016/j.bbamem.2011.12.008. 22197503

[25] Alvarez FJ, Douglas LM, Konopka JB 2007 Sterol-rich plasma membrane domains in fungi. Eukaryot Cell 6:755–763. doi:10.1128/EC.00008-07. 17369440 PMC1899238

[26] Bennion B, Park C, Fuller M, Lindsey R, Momany M, Jennemann R, Levery SB 2003 Glycosphingolipids of the model fungus Aspergillus nidulans: characterization of GIPCs with oligo-alpha-mannose-type glycans. J Lipid Res 44:2073–2088. doi:10.1194/jlr.M300184-JLR200. 12923229

[27] Jorgensen WL, Chandrasekhar J, Madura JD, Impey RW, Klein ML 1983 Comparison of simple potential functions for simulating liquid water. J Chem Phys 79:926–935. doi:10.1063/1.445869.

[28] Miao Y, Sinko W, Pierce L, Bucher D, Walker RC, McCammon JA 2014 Improved reweighting of accelerated molecular dynamics simulations for free energy calculation. J Chem Theory Comput 10:2677–2689. doi:10.1021/ct500090q. 25061441 PMC4095935

[29] Roark M, Feller SE 2009 Molecular dynamics simulation study of correlated motions in phospholipid bilayer membranes. J Phys Chem B 113:13229–13234. doi:10.1021/jp902186f. 19754078

[30] Yue T, Sun M, Zhang S, Ren H, Ge B, Huang F 2016 How transmembranne peptides insert and orient in biomembranes: a combined experimental and simulation study. Phys Chem Chem Phys 18:17483–17494. doi:10.1039/C6CP01133K. 27302083

[31] Lyu Y, Xiang N, Zhu X, Narsimhan G 2017 Potential of mean force for insertion of antimicrobial peptide melittin into a pore in mixed DOPC/DOPG lipid bilayer by molecular dynamics simulations. J Chem Phys 146:155101. doi:10.1063/1.4979613. 28433027

[32] Palma-Guerrero J, Lopez-Jimenez JA, Pérez-Berná AJ, Huang IC, Jansson HB, Salinas J, Villalaín J, Read ND, Lopez-Llorca LV 2010 Membrane fluidity determines sensitivity of filamentous fungi to chitosan. Mol Microbiol 75:1021–1032. doi:10.1111/j.1365-2958.2009.07039.x. 20487294

[33] Phillips JC, Braun R, Wang W, Gumbart J, Tajkhorshid E, Villa E, Chipot C, Skeel RD, Kalé L, Schulten K 2005 Scalable molecular dynamics with NAMD. J Comput Chem 26:1781–1802. doi:10.1002/jcc.20289. 16222654 PMC2486339

[34] Best RB, Zhu X, Shim J, Lopes PEM, Mittal J, Feig M, Mackerell AD 2012 Optimization of the additive CHARMM all-atom protein force field targeting improved sampling of the backbone φ, ψ and side-chain χ(1) and χ(2) dihedral angles. J Chem Theory Comput 8:3257–3273. doi:10.1021/ct300400x. 23341755 PMC3549273

[35] Feller SE, Zhang Y, Pastor RW, Brooks BR 1995 Constant pressure molecular dynamics simulation: the Langevin piston method. J Chem Phys 103:4613–4621. doi:10.1063/1.470648.

[36] Darden T, York D, Pedersen L 1993 Particle mesh Ewald: an N log(N) method for Ewald sums in large systems. J Chem Phys 98:10089–10092. doi:10.1063/1.464397.

[37] van Gunsteren WF, Berendsen HJC 1977 Algorithms for macromolecular dynamics and constraint dynamics. Mol Phys 34:1311–1327. doi:10.1080/00268977700102571.

[38] Jo S, Kim T, Iyer VG, Im W 2008 CHARMM-GUI: a Web-based graphical user interface for CHARMM. J Comput Chem 29:1859–1865. doi:10.1002/jcc.20945. 18351591

[39] Lee J, Cheng X, Swails JM, Yeom MS, Eastman PK, Lemkul JA, Wei S, Buckner J, Jeong JC, Qi Y, Jo S, Pande VS, Case DA, Brooks CL, MacKerell AD, Klauda JB, Im W 2016 CHARMM-GUI input generator for NAMD, GROMACS, AMBER, OpenMM, and CHARMM/OpenMM simulations using the CHARMM36 additive force field. J Chem Theory Comput 12:405–413. doi:10.1021/acs.jctc.5b00935. 26631602 PMC4712441

[40] Klauda JB, Venable RM, Freites JA, O’Connor JW, Tobias DJ, Mondragon-Ramirez C, Vorobyov I, MacKerell AD, Pastor RW 2010 Update of the CHARMM all-atom additive force field for lipids: validation on six lipid types. J Phys Chem B 114:7830–7843. doi:10.1021/jp101759q. 20496934 PMC2922408

[41] Huang J, MacKerell AD 2013 CHARMM36 all-atom additive protein force field: validation based on comparison to NMR data. J Comput Chem 34:2135–2145. doi:10.1002/jcc.23354. 23832629 PMC3800559

[42] Guvench O, Greene SN, Kamath G, Brady JW, Venable RM, Pastor RW, Mackerell AD 2008 Additive empirical force field for hexopyranose monosaccharides. J Comput Chem 29:2543–2564. doi:10.1002/jcc.21004. 18470966 PMC2882059

[43] Le Grand S, Götz AW, Walker RC 2013 SPFP: Speed without compromise - a mixed precision model for GPU accelerated molecular dynamics simulations. Comput Phys Commun 184:374–380. doi:10.1016/j.cpc.2012.09.022.

[44] Loncharich RJ, Brooks BR, Pastor RW 1992 Langevin dynamics of peptides: the frictional dependence of isomerization rates of N‐acetylalanyl‐N′‐methylamide. Biopolymers 32:523–535. doi:10.1002/bip.360320508. 1515543

[45] van den Hondel CAMJJ, Punt PJ, van Gorcom RFM 1991 Heterologous gene expression in filamentous fungi, p 396–418. In Bennett JW, Lasure LL (ed), More gene manipulations in fungi. Academic Press, Inc, San Diego, CA.

[46] Braunschweiler L, Ernst RR 1983 Coherence transfer by isotropic mixing: application to proton correlation spectroscopy. J Magn Reson 53:521–528. doi:10.1016/0022-2364(83)90226-3.

[47] Bax A, Davis DG 1985 MLEV-17-based two-dimensional homonuclear magnetization transfer spectroscopy. J Magn Reson 65:355–360. doi:10.1016/0022-2364(85)90018-6.

[48] Jeener J, Meier BH, Bachmann P, Ernst RR 1979 Investigation of exchange processes by two‐dimensional NMR spectroscopy. J Chem Phys 71:4546–4553. doi:10.1063/1.438208.

[49] Piotto M, Saudek V, Sklenár V 1992 Gradient-tailored excitation for single-quantum NMR spectroscopy of aqueous solutions. J Biomol NMR 2:661–665. doi:10.1007/BF02192855. 1490109

[50] Schanda P, Brutscher B 2005 Very fast two-dimensional NMR spectroscopy for real-time investigation of dynamic events in proteins on the time scale of seconds. J Am Chem Soc 127:8014–8015. doi:10.1021/ja051306e. 15926816

[51] Yuan XL, Roubos JA, Van Den Hondel CAMJJ, Ram AFJ 2008 Identification of InuR, a new Zn(II)2Cys6 transcriptional activator involved in the regulation of inulinolytic genes in Aspergillus niger. Mol Genet Genomics 279:11–26. doi:10.1007/s00438-007-0290-5. 17917744 PMC2129107

[52] Humphrey W, Dalke A, Schulten K 1996 VMD: visual molecular dynamics. J Mol Graph 14:33–38. doi:10.1016/0263-7855(96)00018-5. 8744570

